# Coronary artery bypass grafting in a 14-year-old boy with compound heterozygous LDLR familial hypercholesterolemia: a case report

**DOI:** 10.3389/fped.2025.1625247

**Published:** 2025-12-01

**Authors:** Ke Zhu, Fuqiang Zhang, Jin Wang, Chunjing Li

**Affiliations:** Department of Peking University First Hospital, Beijing, China

**Keywords:** familial hypercholesterolemia, LDLR gene, compound heterozygosity, coronary artery bypass grafting, pediatric cardiovascular disease

## Abstract

Familial hypercholesterolemia (FH), particularly homozygous or compound heterozygous forms, predisposes individuals to premature cardiovascular disease due to severely elevated low-density lipoprotein cholesterol (LDL-C). This case report describes a 14-year-old boy with compound heterozygous pathogenic variants in the LDLR gene, diagnosed with FH with a strong family history of hypercholesterolemia. Despite early initiation of statins, the patient developed progressive angina pectoris. Coronary angiography revealed critical stenosis in the left main arteries, necessitating urgent coronary artery bypass grafting (CABG). Arterial as opposed to venous conduit selection in pediatric homozygous familial hypercholesterolemia warrants explicit rationale. In the postoperative stage, LDL-C levels remained elevated but were managed with adjunctive therapies, including PCSK9 inhibitor. Genetic testing confirmed compound heterozygosity, underscoring the aggressive nature of LDLR dysfunction. This case highlights the challenges of managing severe FH in pediatric patients, emphasizing the importance of early genetic diagnosis, multimodal lipid-lowering therapy, and timely surgical intervention to prevent life-threatening complications. It also reinforces the necessity of lifelong monitoring and developing novel therapeutic strategies to treat compound heterozygous FH cases. This report contributes to the limited literature on CABG in pediatric FH, advocating for a multidisciplinary approach to optimize outcomes in this high-risk population.

## Introduction

Familial hypercholesterolemia (FH) is an inherited metabolic disorder characterized by mutation in low-density lipoprotein (LDL) receptor gene, which causes severely elevated LDL cholesterol levels (1). It leads to a markedly increased risk of coronary heart disease (CHD) and the development of multiple xanthomas, with variable severity depending on the causative mutation (1, 2). According to the new clinical classification proposed by Luis Masana in 2019, FH can be divided into the following four types: heterozygous familial hypercholesterolemia (HeFH), homozygous familial hypercholesterolemia (HoFH), polygenic familial hypercholesterolemia, and familial hypercholesterolemia combined with hypertriglyceridemia (3). HeFH is a frequent monogenic hereditary disorder with a prevalence of approximately 1 in 250 to 1 in 300; conversely, HoFH is a rare condition with a prevalence of approximately 1 in 250,000 to 1 in 360,000 (4–6). However, compound heterozygous FH (cHeFH), a kind of HoFH, is even more uncommon (7). Based on the latest clinical guidelines (8), a phenotypic diagnosis of HoFH or HeFH should be prioritized over genetic findings, with diagnostic thresholds updated as per EAS 2023 (8) criteria—where an LDL-C level exceeding 10 mmol/L suggests HoFH, irrespective of genotype.

Herein, we present the case of a young patient with cHeFH who presented with chest pain during exercise caused by coronary arteries stenosis and underwent coronary artery bypass graft (CABG) surgery at the age of 14 years.

## Case presentation

A 14-year-old boy was admitted to the Pediatric Intensive Care Unit of Peking University First Hospital in July 2023 with the primary complaint of chest pain after exercise and xanthomas. Unexpectedly, the child had experienced multiple xanthomas over the previous 5 years. He began to experience chest pain 3 years ago when he was running or jumping. He also experienced a stabbing pain in the precordial area that was relieved after 1–2 min of rest. At the same time, there was documented evidence of hypercholesterolemia with a total cholesterol level of 17.37 mmol/L and an LDL cholesterol level of 14.23 mmol/L. He had previously received lipid-lowering therapy, including atorvastatin and some Chinese herbal medications (the specific duration and dosage could not be specified), but no significant improvement was observed.

In June 2020, treatment with atorvastatin (20 mg once daily) was initiated. However, the medication was self-discontinued after approximately 1 year due to lack of significant lipid improvement (exact date unknown). In December 2021, coronary CTA was performed at another hospital, revealing no significant plaque or stenosis in the right coronary artery, left main coronary artery, left anterior descending branch, or obtuse marginal branch. The patient subsequently self-administered rosuvastatin (20 mg once daily) without medical consultation (exact start date unknown). In June 2022, follow-up lipid testing showed markedly elevated levels of total cholesterol at 15.98 mmol/L and low-density lipoprotein cholesterol at 14.37 mmol/L, after which rosuvastatin was again self-discontinued (exact date unknown).

## Medical history and family history

The patient had no significant past medical history and denied any history of hypertension, diabetes, renal disease, thyroid dysfunction, autoimmune disorders, or Kawasaki disease. He was the second child, born full term via spontaneous vaginal delivery, with no history of perinatal hypoxia or asphyxia. Since early childhood, his weight and height had consistently remained below those of his peers, although his intellectual capabilities and motor development were age appropriate.

There is a notable family history of dyslipidemia: the patient's father, grandfather, uncle, and elder sister all exhibited lipid abnormalities. Specific total cholesterol and low-density lipoprotein cholesterol (LDL-C) levels for the father, grandfather, and uncle are unavailable. The patient's father died suddenly at age 37 without prior evaluation for atherosclerotic cardiovascular disease (ASCVD). The patient's uncle also died suddenly at age 32; he had been diagnosed with coronary artery disease at approximately 28 years and underwent coronary artery bypass grafting. The patient's 17-year-old sister had the following lipid profile: total cholesterol: 8.32 mmol/L; LDL-C: 5.47 mmol/L; and high-density lipoprotein cholesterol (HDL-C): 1.34 mmol/L. The patient's 41-year-old mother had the following lipid levels: total cholesterol: 4.7 mmol/L; LDL-C: 3.23 mmol/L; and HDL-C: 1.01 mmol/L.

After excluding other conditions associated with elevated LDL-C levels, genetic testing was performed, and the patient was diagnosed with FH with compound heterozygous variation of LDLR gene. Two LDLR gene variants (NM_000527.4) were identified: c.1864G>T/p.Asp622Tyr (paternal origin) and c.1474G>A/p.Asp492Asn (maternal origin). Segregation analysis confirmed that the p.Asp622Tyr variant was inherited from the father, who had a clinical history of severe hypercholesterolemia, while the p.Asp492Asn variant was inherited from the mother, who also exhibited a hypercholesterolemic phenotype. The proband is a compound heterozygote for these two pathogenic LDLR variants.

Consistent with the 2023 guidance from the European Atherosclerosis Society (8) (EAS), the diagnosis of HoFH in this proband is primarily driven by the profound clinical phenotype, characterized by significantly elevated LDL-C levels from childhood and the presence of cutaneous xanthomas. Genetically, the proband is a compound heterozygote for two pathogenic LDLR variants. This genotype is consistent with a severe FH phenotype. The p.Asp622Tyr and p.Asp492Asn variants are both located in the epidermal growth factor (EGF) precursor homology domain of the LDLR protein, a region critical for receptor recycling. Defects in this domain typically result in a recycling-deficient receptor, leading to a significant reduction in LDL clearance capacity.

On admission to our ward, the patient was 146 cm tall, weighed 28.2 kg (body mass index 13.22 kg/m^2^), and displayed symptoms of protein-energy malnutrition (PEM). A physical examination revealed the following data: T: 37.0 °C; P: 115 beats per minute; R: 20 times per minute; and blood pressure: 127/71 mmHg. There were multiple xanthomas around the tendons of his elbows and patellar, with a maximum diameter of 20 mm, without ulceration or itching. On cardiac examination, a grade 2–3/6 murmur, P2 > A2, was heard in the precordial area, and a systolic murmur was heard bilaterally in the neck and at the upper border of the right clavicle. Moreover, lipid corneal arch was seen.

The relevant laboratory investigations regarding the serum lipid levels of the patient are detailed in the Table 1.

**Table 1:** Patient's serum lipid levels.

Time	TC (mmol/L)	Triglyceride (mmol/L)	HDL-C (mmol/L)	LDL-C (mmol/L)	Lpp A (mg/L)	Apo A g/L	Apo B g/L
4 June 2020	15.61	/	/	12.41	/	/	/
6 June 2020	15.30	/	/	11.92	313	/	2.98
15 June 2020	17.37	/	0.81	14.21	576	/	3.49
15 July 2020	17.37	/	/	14.23	/	/	/
29 June 2022	15.98	/	/	14.37	/	/	/
19 July 2023	16.34	0.73	0.95	13.92	320.10	0.99	>2.0
28 July 2023	14.39	0.9	0.75	12.12	/	/	/
4 August 2023	13.67	1.25	0.78	11.45	831.30	0.92	>2.0
8 August 2023	11.97	0.82	0.71	9.53	/	/	/
21 August 2023 (the 10th day after the operation)	6.11	1.16	0.56	4.92	/	/	/
21 September 2023 (1 month after the operation)	9.26	/	/	6.87	/	/	/
August 2024 (1 year after the operation)	<7	/	/	/	/	/	/

TC, total cholesterol; HDL-C, high-density lipoprotein cholesterol; LDL-C, low-density lipoprotein cholesterol; Lpp A, lipoprotein A; ApoA, apolipoprotein A; ApoB, apolipoprotein B.

**Figure 1:**
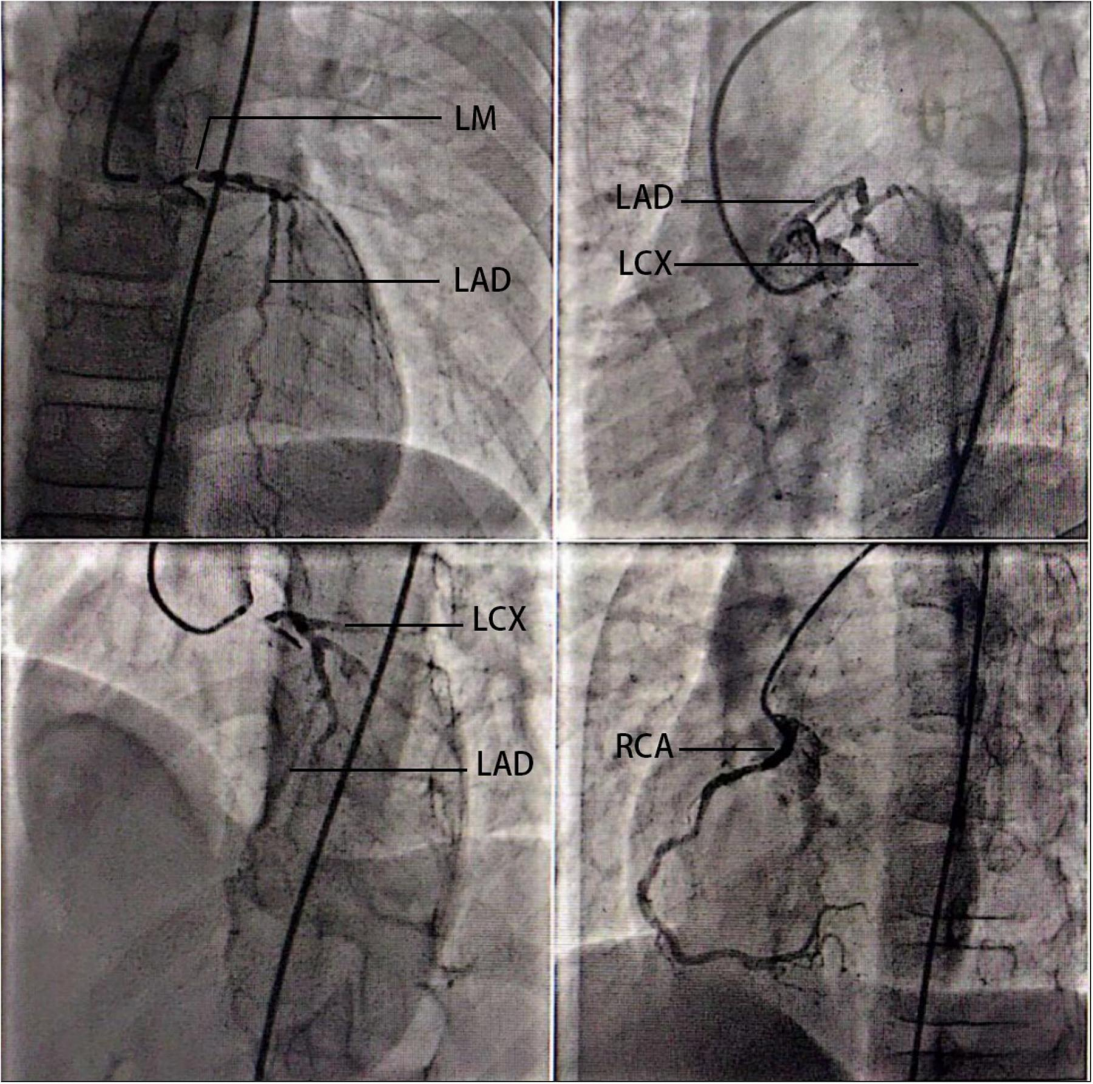
Coronary angiography of the 14-year-old patient. Coronary angiography (27 July 2023) demonstrated significant left main coronary artery disease with 85% stenosis, along with 30% stenosis in the proximal left anterior descending artery, 30% stenosis in the distal circumflex artery, and 40% stenosis in the proximal right coronary artery. LM, left main coronary artery; LAD, left anterior descending artery; LCX, left circumflex artery; RCA, right coronary artery.

In the final preoperative assessment (8 August 2023), transthoracic echocardiography revealed a left ventricular ejection fraction of 72.2%, left ventricular (LV) enlargement (LV end-diastolic volume 57.0 mL, LV end-systolic volume 15.8 mL), mild supravalvular aortic stenosis, mild aortic regurgitation, and mild tricuspid regurgitation. Coronary angiography (27 July 2023) demonstrated significant left main coronary artery disease with 85% stenosis, along with 30% stenosis in the proximal left anterior descending artery, 30% stenosis in the distal circumflex artery, and 40% stenosis in the proximal right coronary artery (Figure 1). A carotid Doppler ultrasound revealed an increased carotid artery intima-media thickness (cIMT) as well as signs of atherosclerosis. Plaque formation occurred in bilateral common carotid arteries, bilateral internal carotid arteries, left external carotid arteries, right vertebral arteries, and right subclavian arteries. Holter monitoring demonstrated mild ST-T segment changes after exercise, and the NT-ProBNP level was 234 pg/mL.

During hospitalization, the patient was initiated on a combination pharmacotherapy regimen. Lipid-lowering therapy included atorvastatin (10 mg nightly starting on 20 July 2023, gradually increased to 40 mg nightly, and discontinued on 30 July), followed by rosuvastatin (20 mg nightly starting on 1 August), in combination with ezetimibe (10 mg daily starting on 24 July 2023) and evolocumab (140 mg biweekly starting on 28 July 2023). Antiplatelet therapy included aspirin (100 mg daily starting on 21 July). For heart rate control, metoprolol was initiated (6.25 mg twice daily starting on 21 July 2023, gradually uptitrated to 12.5 mg twice daily). In addition, a nutrition consultation was arranged for dietary guidance. Meanwhile, a multidisciplinary consultation was conducted for this patient, involving cardiology, cardiac surgery, pediatric surgery, anesthesiology, and the pediatric intensive care unit. Based on a discussion with the concerned specialists, a treatment plan was formulated to continue the current medical management while considering coronary artery bypass grafting. The procedure was performed under general anesthesia with endotracheal intubation. Preoperative laboratory tests (8 August 2023) revealed that his total cholesterol (TC) level had decreased to 11.97 mmol/L, with a low-density lipoprotein (LDL) level of 9.53 mmol/L. Intraoperative monitoring included electrocardiogram, pulse oxygen saturation, invasive blood pressure, transesophageal echocardiography (TEE), central venous catheterization (CVC), and arterial blood gas (ABG) analysis. Cardiopulmonary bypass was employed during operation.

Under general anesthesia, the cardiac surgeons opened the chest via a midline incision. After establishing cardiopulmonary bypass, the midsegment of the left anterior descending artery (LAD) and the smaller-diameter circumflex artery were incised, and end-to-side anastomosis was performed with the great saphenous vein. However, the blood flow was found to be unsatisfactory. Next, the intermediate branch was incised and an end-to-side anastomosis was performed with the great saphenous vein. Then, the surgeons performed end-to-side anastomoses of the proximal ends of the great saphenous vein grafts to the ascending aorta. The aortic punch holes for the proximal anastomoses measured 3 mm in diameter. The exact internal diameters of the venous grafts were not specified in the operative records. Following cardiac reperfusion, an end-to-side Y-type anastomosis was performed between the saphenous vein graft anastomosed to the intermediate branch and the graft anastomosed to the circumflex artery. Finally, the surgeons stopped the cardiopulmonary bypass and performed a continuous suture on the incision.

The postoperative course in the ICU was uneventful and the patient was transferred back to the general ward on the day following the operation. The patient's postoperative regimen included dual antiplatelet therapy (DAPT: aspirin 100 mg qd, later reduced to 50 mg qd; clopidogrel 25 mg bid) in addition to his ongoing lipid-lowering therapy. He was discharged after 12 days following an unremarkable recovery. His total cholesterol levels were 5.24 mmol/L at their lowest (17 August 2023) and 6.11 mmol/L upon discharge (21 August 2023). He was dispensed the following medications upon discharge: Triple lipid-lowering therapy involved rosuvastatin (20 mg qn), ezetimibe (10 mg qd), and a PCSK9 inhibitor, while DAPT involved clopidogrel (25 mg bid) and aspirin (50 mg qd).

During the postoperative follow-up, the patient reported no complaints of chest pain, chest tightness, palpitations, or dizziness after discharge and adhered to regular medication. Laboratory tests at the 1-month follow-up (11 September 2023) revealed a total cholesterol level of 9.26 mmol/L and a low-density lipoprotein cholesterol level of 6.87 mmol/L. During the 1-year telephone follow-up, the family reported that the patient remained free of myocardial ischemia-related symptoms, continued with pharmacotherapy, and had achieved a total cholesterol level below 7 mmol/L. Liver transplantation has not been performed for the patient.

The treatment process of the patient after admission is detailed in Table 2.

**Table 2:** Timeline of the patient's treatment course.

Date	Category of intervention	Detailed description
20 July 2023	Lip-lowering therapy (initiation, phase 1)	Atorvastatin was initiated at an initial dose of 10 mg once nightly (qn); the dose was gradually uptitrated to 40 mg qn thereafter.
21 July 2023	Antiplatelet therapy + heart rate control	1. Antiplatelet therapy: Aspirin [100 mg once daily (qd)] was administered. 2. Heart rate control: Metoprolol was initiated at 6.25 mg twice daily (bid), with subsequent uptitration to 12.5 mg bid.
24 July 2023	Lip-lowering therapy (regimen adjustment)	Ezetimibe (10 mg qd) was added to the existing atorvastatin therapy, forming a dual lipid-lowering regimen.
27 July 2023	Multidisciplinary consultation (MDC)	Following coronary angiography, a hospital-wide MDC was convened, involving specialists from Cardiology, Cardiac Surgery, Anesthesiology, Pediatric Surgery, Pediatric Critical Care Medicine, and Pediatric Cardiology. Coronary artery bypass grafting (CABG) was recommended for the pediatric patient.
28 July 2023	Lip-lowering therapy (triple regimen optimization)	Evolocumab was initiated at a dose of 140 mg per administration, once every 2 weeks (q2w). This agent was combined with atorvastatin and ezetimibe to form a triple lipid-lowering regimen.
30 July 2023	Lip-lowering therapy (drug discontinuation)	Atorvastatin was discontinued due to subsequent regimen adjustment.
1 August 2023	Lip-lowering therapy (drug replacement)	Rosuvastatin Calcium Tablets were initiated at 20 mg qn, replacing atorvastatin; the agent was continued in combination with ezetimibe and evolocumab to maintain the triple lipid-lowering effect.
7 August 2023	Preoperative management and ward transfer	After thorough communication with the patient's guardians and optimization of preoperative treatment, the patient was transferred to the Cardiac Surgery Ward to complete preoperative preparation.
11 August 2023	Surgical intervention (CABG)	The patient underwent CABG under general anesthesia (GA): 1. Graft vessel: Great saphenous vein (GSV); 2. Target vessels for bypass: Left anterior descending artery (LAD), intermediate branch (IB), and circumflex branch (CX); 3. Intraoperative outcome: The procedure was completed uneventfully.
11–23 August 2023	Postoperative care and recovery	1. Supportive care: Respiratory support, packed red blood cell (pRBC) transfusion; 2. Prophylactic intervention: Cefotiam for infection prophylaxis, omeprazole for gastric mucosal protection; 3. Symptomatic treatment: Isosorbide Mononitrate Tablets for symptomatic relief; 4. Chronic therapy continuation: Triple lipid-lowering therapy (rosuvastatin + ezetimibe + evolocumab) was maintained; dual antiplatelet therapy (DAPT) (clopidogrel + aspirin) was added; 5. Recovery status: The patient achieved uneventful recovery with no subjective complaints.
23 August 2023	Discharge	The patient was discharged from the hospital in stable condition.
11 September 2023	Postdischarge follow-up [(1 month after the operation)]	1. Patient-reported outcome: No complaints of chest pain, chest tightness, palpitations, dizziness, or other adverse symptoms; medication adherence was confirmed. 2. Laboratory findings: Serum total cholesterol (TC) 9.26 mmol/L, low-density lipoprotein cholesterol (LDL-C) 6.87 mmol/L.
August 2024	Postdischarge follow-up (1 year after the operation)	On telephone follow-up, the family reported that the patient remained free of myocardial ischemia-related symptoms, continued on pharmacotherapy, and had achieved a total cholesterol level below 7 mmol/L.

**Figure 2:**
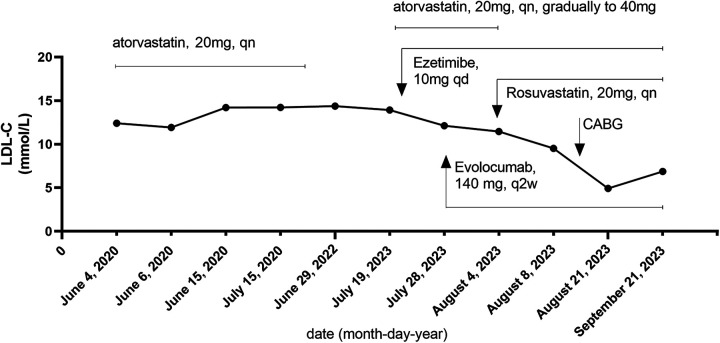
LDL-C trajectory.

Furthermore, the LDL-C trajectory is presented in Figure 2, aligned to interventions. The longitudinal LDL-C trajectory depicted in Figure 2 illustrates the dynamic response to sequential therapeutic interventions, including high-intensity statin therapy, ezetimibe, and PCSK9 inhibitors. Furthermore, it should be noted that the patient initially self-administered atorvastatin (20 mg, qn) in June 2020, following the detection of severe hyperlipidemia (TC: 15.61 mmol/L, LDL-C: 12.41 mmol/L).

Due to a perceived lack of significant improvement in lipid levels after approximately 1 year, the medication was independently discontinued by the patient. Upon presentation to our hospital in July 2023, atorvastatin was reinitiated at 20 mg qn and subsequently uptitrated to 40 mg qn. As the therapeutic response remained suboptimal, the patient was switched to the more potent agent rosuvastatin (20 mg, qn).

## Discussion

HoFH is a rare autosomal semidominant disorder with equal gender distribution (8). It is subclassified into four categories: (a) true homozygous, characterized by identical mutations in both alleles of an FH-related gene (LDLR, APOB, or PCSK9 inhibitor); (b) compound heterozygous (cHeFH), involving two different mutations affecting the same gene on different alleles; (c) combined heterozygous, resulting from mutations in two different FH-associated genes; and (d) autosomal recessive hypercholesterolemia (ARH), which presents an HoFH phenotype but follows an autosomal recessive inheritance pattern (3).

cHeFH is a rare disorder resulting from disease-causing variants in both alleles of the LDLR or other FH-related genes. It is characterized by markedly elevated levels of LDL-C from conception and accelerated ASCVD, often resulting in early death (8, 9). A combination therapy of statins and ezetimibe is efficient to decrease the risk of severe cardiovascular events; however, this therapy cannot reverse the vascular stenosis, calcified changes, and chronic ischemic lesions. Timely revascularization is a critical component of management for symptomatic patients with significant coronary artery disease, alongside intensive LDL-C-lowering therapy (8).

### Heart team rationale for favoring CABG over PCI

The heart team’s rationale for favoring CABG over PCI was based on the patient's unique age and anatomical considerations. Given the pediatric status of the patient, the coronary arteries were of smaller caliber and subject to ongoing physiological growth. A rigid stent implant cannot accommodate the natural increase in coronary artery diameter over time, posing a significant risk of future relative stent stenosis. In addition, the child presented with severe stenosis of the left main coronary artery, unstable plaque, and involvement of other coronary arteries, which collectively met the indications for pediatric CABG. Accordingly, CABG surgery was considered the best option to alleviate the symptoms.

We reported the case of a 14-year-old boy with cHeFH and mutations in LDLR. He fulfilled the FH Dutch diagnostic criteria (10) for definite FH. On account of the prognosis of coronary heart disease and the worsening of symptoms following physical effort, CABG was performed.

However, the implementation of cardiac surgery and anesthesia in a 14-year-old child with PEM undoubtedly posed grave challenges due to which perioperative management was particularly important.

### Assess and correct the status of malnutrition before cardiac surgery

Previous studies have reported that patients benefit from an assessment of their nutritional status and supplemental management to address insufficient protein and caloric intake before undergoing cardiac surgery (11, 12).

### Maintain hemodynamic stability

A strategy of hemodynamic optimization was adopted, which included the systematic monitoring of cardiac output, goal-directed therapy, and the use of vasoactive agents. Moreover, the dosage of anesthesia was adjusted dynamically according to the surgical procedure.

### Protective ventilation strategy

A protective ventilation strategy was utilized during CABG surgery to reduce the incidence of postoperative pulmonary complications and length of stay in hospital, as recommended (13–15). Our intraoperative mechanical ventilation strategy was aligned with lung-protective principles recommended for cardiac surgery (16). In particular, we utilized a low tidal volume of 6–8 mL/kg of the predicted body weight and a positive end-expiratory pressure (PEEP) of 5 cmH_2_O. Alveolar recruitment maneuvers were performed upon weaning from cardiopulmonary bypass (CPB), sustained at an airway pressure of 30–35 cmH_2_O for 10 s. This approach could improve oxygenation and reduce atelectasis after cardiac surgery.

### Early tracheal extubation

The safety of early tracheal extubation was confirmed in a large-scale study (17). Early extubation could reduce the occurrence of postoperative complications and the length of stay in the intensive care unit and hospital (18). However, caution is advised in high-risk patients. In our case, the endotracheal tube of the patient was extubated on the first postoperative day, given his status. Extubation was performed only upon meeting standardized criteria: hemodynamic stability without significant vasopressor support; adequate gas exchange (PaO_2_/FiO_2_ > 200 on minimal ventilator settings); normal core temperature; absence of excessive bleeding (<100 mL/h); and patient consciousness with the ability to respond to commands.

### Analgesia in cardiac surgery

An opioid-sparing analgesic strategy was employed, and postoperative patient-controlled analgesia was performed. Adequate analgesia was ensured to avoid stress response and aggravation of cardiac conditions. Our perioperative care incorporated key elements, such as preoperative patient education and counseling, multimodal analgesia to minimize opioid consumption, and early de-escalation of invasive monitoring and early mobilization (within 24 h following extubation, as tolerated).

### Venous graft

Preoperative vascular ultrasonography indicated elevated blood flow velocities in both subclavian arteries, raising the suspicion of potential subclavian artery stenosis. Given the patient's diagnosis of HoFH, which carries an extreme risk of aggressive and systemic atherosclerosis, the team was concerned about future disease progression. Using an *in situ* left ITA (LITA) graft to the LAD would carry a significant long-term risk of “coronary steal” and graft failure should a hemodynamically significant stenosis develop proximally in the subclavian artery. This was deemed an unacceptable risk. Furthermore, the literature supports that SVGs are a reasonable and effective alternative in specific pediatric populations, particularly when the arterial system is compromised. Our decision is supported by case series and reports (19), which indicate that SVGs in children can demonstrate acceptable growth potential and long-term patency in conditions such as Kawasaki disease. This provides a rationale for their use when arterial grafts are not feasible.

Furthermore, several patient-specific factors reinforced this choice. This 14-year-old patient had lesions in most of his arteries, yet preoperative evaluation confirmed that his great saphenous vein was of good quality and free from atheromatous plaques. In addition, the patient was a potential candidate for future liver transplantation—a procedure that could address the underlying metabolic disorder. Thus, the venous graft was considered a justifiable bridging solution. Previous studies have reported acceptable outcomes with venous grafts in similar contexts (20, 21). Fortunately, the procedure was completed successfully, and the patient's intraoperative and postoperative courses were uneventful.

### Medication

The patient recovered well after surgery with triple lipid-lowering therapy and dual antiplatelet therapy, which provided relief from symptoms, clinically improved cardiovascular disorders, and enhanced exercise tolerance.

In terms of the expected efficacy of evolocumab in pediatrics, we provided clarification on its regulatory context. It is currently approved in several regions for the treatment of HeFH in patients aged 10 years and older. In addition, data in pediatric HoFH patients (22) demonstrate its potential for LDL-C reduction.

The expected efficacy of evolocumab (a PCSK9 inhibitor) is highly dependent on the underlying LDLR genotype. In patients with defective LDLR mutations (residual function), the drug can produce a substantial reduction in LDL-C (up to 40%–60%) by blocking PCSK9-mediated degradation of the functional LDLRs. In contrast, for patients with LDLR-negative genotypes (null alleles), who completely lack functional LDLRs, the expected efficacy of evolocumab is minimal to absent.

The long-term safety and efficacy of evolocumab in the management of familial hypercholesterolemia in pediatric patients will require further study.

### LDL-C management strategy and attainment of guideline-recommended targets

In accordance with contemporary lipid-lowering standards (8), we aligned our lipid-lowering strategy with the recommended LDL-C targets for patients with homozygous or compound heterozygous familial hypercholesterolemia (HoFH/cHeFH). In children and adolescents, an LDL-C goal of <3 mmol/L (<115 mg/dL) is recommended if treatment is initiated before 18 years and imaging assessment does not indicate ASCVD, with a lower goal in those with established ASCVD. Despite intensive multidrug therapy, a significant proportion of patients failed to attain the predefined LDL-C targets, primarily due to be the severity of the underlying LDL receptor (LDLR) dysfunction and the limited efficacy of conventional therapies, including PCSK9 inhibitors, in patients with null–null LDLR mutations.

With regard to adjunctive therapies, the use of lomitapide (an MTP inhibitor) or evinacumab (an ANGPTL3 inhibitor) was considered for eligible patients with refractory hypercholesterolemia. However, their availability and implementation were constrained by local regulatory approval status and reimbursement policies at the time of the study. Data for these newer agents are, however, limited in children with HoFH.

LDL apheresis has long served as a cornerstone therapy for many severe cases, effectively providing acute reductions in LDL-C. Lipoprotein apheresis is foundational in children and adults with HoFH, functioning as an adjunct to other lipid-lowering therapy.60 It is therefore vital in countries with limited access to newer therapies. Limitations include variable access, cost, and a time-consuming procedure, all of which negatively impact the patient's quality of life.

Liver transplantation may be an option for a small subset of patients with HoFH, particularly severely affected young children with biallelic null variants. The benefits of liver transplantation need to be balanced against the risks.

Novel approaches to PCSK9 and ANGPTL3 blockade, including small interfering (si)RNA therapies, are currently in development. Advances in biotechnology offer the future possibility of liver-directed gene transfer and gene editing for HoFH. Although promising, these approaches require careful evaluation of intermediate and long-term safety and efficacy.

### Limitations

The current study has certain limitations. First, despite our best efforts, the patient and his immediate family members were unwilling to provide detailed pedigree information and their personal lipid level data due to privacy concerns. We have, however, included all available family history information as reported by the patient. Second, the 1-year postoperative follow-up was conducted via telephone with the patient's family. We fully acknowledge the inherent limitation of this method for precise data collection, including the possibility of individual variability, challenges with long-term medication adherence, and the limitations of remote follow-up. This adds an important clinical perspective to the challenges of managing severe familial hypercholesterolemia. In addition, the exact internal diameters of the venous grafts were not specified in the operative records. In terms of graft patency, systematic, protocol-driven imaging (such as CT angiography or Doppler echocardiography) at 6–12 months following intervention was not routinely performed as part of the standard care for the patients in this retrospective cohort and is therefore unavailable for analysis. We recognize the absence of this objective data as a limitation of our study, as it restricts our ability to fully evaluate the long-term structural integrity of the surgical intervention. Concerning growth metrics, the systematic collection and documentation of serial weight and height measurements, necessary for calculating precise z-scores, were not consistently available in the medical records for all patients before and after operation. Furthermore, a formal patient perspective was not obtained in this case, which is another limitation.

In the follow-up 1 year after the CABG, there were no signs of ischemia. The patient recovered well and was ready for further treatment.

## Innovation and key contributions

1. Rare Pediatric Case: We report coronary artery bypass grafting (CABG) performed in a 14-year-old boy with compound heterozygous LDLR variants, a genetic profile associated with aggressive homozygous FH. The manifestation of this condition in pediatric patients requiring CABG is exceedingly rare, emphasizing the urgency of early diagnosis and intervention.
2. Genetic and Clinical Correlations: The patient had compound heterozygous mutations in LDLR (NM_000527.4)—c.1864G>T/p.Asp622Tyr (paternal origin) and c.1474G>A/p.Asp492Asn (maternal origin)—which led to a significant increase in LDL, accelerated atherosclerosis, and multivessel coronary stenosis. Our report provides detailed genotypic-phenotypic correlations, underscoring the role of genetic testing in guiding clinical decisions for refractory hypercholesterolemia.
3. Surgical Challenges and Outcomes: CABG in pediatric patients presents unique challenges, including graft selection, management of calcified vessels, and postoperative care.
4. Perioperative Anesthetic Challenges: The perioperative management of adolescents undergoing CABG presents a multidimensional challenge encompassing considerations of low body weight, age-specific anesthetic requirements, and inherent risks of coronary revascularization, particularly given the critical implications of proximal lesion localization. Key anesthetic strategies include analgesia optimization, hemodynamic stability protocol, early postoperative extubation, and enhanced recovery pathway. This structured approach addresses the unique physiological requirements of adolescent cardiac patients while optimizing surgical outcomes through targeted perioperative interventions.

## Data Availability

The original contributions presented in the study are included in the article/Supplementary Material, further inquiries can be directed to the corresponding author.

## References

[1] Singh S Bittner V. Familial hypercholesterolemia—epidemiology, diagnosis, and screening. Curr Atheroscler Rep. (2015) 17(2):482. 10.1007/s11883-014-0482-5 25612857

[2] Sánchez-Hernández RM Prieto-Matos P Civeira F Lafuente EE Vargas MF Real JT Autosomal recessive hypercholesterolemia in Spain. Atherosclerosis. (2018) 269:1–5. 10.1016/j.atherosclerosis.2017.12.006 29245109

[3] Masana L Ibarretxe D Rodríguez-Borjabad C Plana N Valdivielso P Pedro-Botet J Toward a new clinical classification of patients with familial hypercholesterolemia: one perspective from Spain. Atherosclerosis. (2019) 287:89–92. 10.1016/j.atherosclerosis.2019.06.905 31238171

[4] Hu P Dharmayat KI Stevens CAT Sharabiani MTA Jones RS Watts GF Prevalence of familial hypercholesterolemia among the general population and patients with atherosclerotic cardiovascular disease. Circulation. (2020) 141(22):1742–59. 10.1161/CIRCULATIONAHA.119.044795 32468833

[5] Beheshti SO Madsen CM Varbo A Nordestgaard BG. Worldwide prevalence of familial hypercholesterolemia. J Am Coll Cardiol. (2020) 75(20):2553–66. 10.1016/j.jacc.2020.03.057 32439005

[6] Defesche JC Gidding SS Harada-Shiba M Hegele RA Santos RD Wierzbicki AS. Familial hypercholesterolaemia. Nat Rev Dis Primers. (2017) 3(1):17093. 10.1038/nrdp.2017.93 29219151

[7] Sjouke B Kusters DM Kindt I Besseling J Defesche JC Sijbrands EJG Homozygous autosomal dominant hypercholesterolaemia in the Netherlands: prevalence, genotype–phenotype relationship, and clinical outcome. Eur Heart J. (2015) 36(9):560–5. 10.1093/eurheartj/ehu058 24585268

[8] Cuchel M Raal FJ Hegele RA Al-Rasadi K Arca M Averna M 2023 update on European Atherosclerosis Society Consensus Statement on Homozygous Familial Hypercholesterolaemia: new treatments and clinical guidance. Eur Heart J. (2023) 44(25):2277–91. 10.1093/eurheartj/ehad197 37130090 PMC10314327

[9] Marusic T Sustar U Sadiq F Kotori V Mlinaric M Kovac J Genetic and clinical characteristics of patients with homozygous and compound heterozygous familial hypercholesterolemia from three different populations: case series. Front Genet. (2020) 11:572176. 10.3389/fgene.2020.572176 33093846 PMC7528874

[10] Nordestgaard BG Chapman MJ Humphries SE Ginsberg HN Masana L Descamps OS Familial hypercholesterolaemia is underdiagnosed and undertreated in the general population: guidance for clinicians to prevent coronary heart disease: consensus statement of the European Atherosclerosis Society. Eur Heart J. (2013) 34(45):3478–90. 10.1093/eurheartj/eht273 23956253 PMC3844152

[11] Chambrier C Sztark F. Recommandations de bonnes pratiques cliniques sur la nutrition périopératoire. Actualisation 2010 de la conférence de consensus de 1994 sur la « nutrition artificielle périopératoire en chirurgie programmée de l’adulte ». Ann Françaises Anesth Réanimation. (2011) 30(4):381–9. 10.1016/j.annfar.2011.01.014 21435816

[12] Wischmeyer PE Carli F Evans DC Guilbert S Kozar R Pryor A American Society for enhanced recovery and perioperative quality initiative joint consensus statement on nutrition screening and therapy within a surgical enhanced recovery pathway. Anesth Analg. (2018) 126(6):1883–95. 10.1213/ANE.0000000000002743 29369092

[13] Mathis MR Duggal NM Likosky DS Haft JW Douville NJ Vaughn MT Intraoperative mechanical ventilation and postoperative pulmonary complications after cardiac surgery. Anesthesiology. (2019) 131(5):1046–62. 10.1097/ALN.0000000000002909 31403976 PMC6800803

[14] Leme AC Hajjar LA Volpe MS Fukushima JT Santiago RRDS Osawa EA Effect of intensive vs moderate alveolar recruitment strategies added to lung-protective ventilation on postoperative pulmonary complications: a randomized clinical trial. JAMA. (2017) 317(14):1422–32. 10.1001/jama.2017.2297. 28322416

[15] Engelman DT Ali WB Williams JB Perrault LP Reddy VS Arora RC Guidelines for perioperative care in cardiac surgery: enhanced recovery after surgery society recommendations. JAMA Surg. (2019) 154(8):755–66. 10.1001/jamasurg.2019.1153 31054241

[16] Zochios V Klein AA Gao F. Protective invasive ventilation in cardiac surgery: a systematic review with a focus on acute lung injury in adult cardiac surgical patients. J Cardiothorac Vasc Anesth. (2018) 32(4):1922–36. 10.1053/j.jvca.2017.10.031 29199052

[17] Richey M Mann A He J Daon E Wirtz K Dalton A Implementation of an early extubation protocol in cardiac surgical patients decreased ventilator time but not intensive care unit or hospital length of stay. J Cardiothorac Vasc Anesth. (2018) 32(2):739–44. 10.1053/j.jvca.2017.11.007 29229252

[18] Mertes P-M Kindo M Amour J Baufreton C Camilleri L Caus T Guidelines on enhanced recovery after cardiac surgery under cardiopulmonary bypass or off-pump. Anaesth Crit Care Pain Med. (2022) 41(3):101059. 10.1016/j.accpm.2022.101059 35504126

[19] Suda Y Takeuchi Y Ban T Ichikawa S Higashita R. Twenty-two-year follow-up of saphenous vein grafts in pediatric Kawasaki disease. Ann Thorac Surg. (2000) 70(5):1706–8. 10.1016/s0003-4975(00)01374-6 11093521

[20] U Ersoy MG. Coronary revascularization in a seven-year-old boy with homozygous familial hypercholesterolaemia. Acta Paediatr 2020. (2000) 89(12):1501–2. 10.1080/080352500456750 11195247

[21] El-Khouri HM Danilowicz DA Slovis AJ Colvin SB Artman M. Saphenous vein graft growth 13 years after coronary bypass in a child with Kawasaki disease. Ann Thorac Surg. (1998) 65(4):1127–30. 10.1016/S0003-4975(97)01415-X 9564940

[22] Santos RD Ruzza A Hovingh GK Wiegman A Mach F Kurtz CE Evolocumab in pediatric heterozygous familial hypercholesterolemia. N Engl J Med. (2020) 383(14):1317–27. 10.1056/NEJMoa2019910 32865373

